# Comprehensive Analysis of Opioid Use After Common Elective Outpatient Orthopaedic Surgeries

**DOI:** 10.5435/JAAOSGlobal-D-21-00226

**Published:** 2022-04-12

**Authors:** Michael U. Okoli, Alexander J. Rondon, Clay B. Townsend, Matthew B. Sherman, Asif M. Ilyas

**Affiliations:** From the Rothman Orthopaedic Institute at Thomas Jefferson University, Philadelphia, PA (Dr. Okoli, Dr. Rondon, Dr. Townsend, Dr. Sherman, and Dr. Ilyas), and the Rothman Opioid Foundation, Philadelphia, PA (Dr. Ilyas).

## Abstract

**Background:**

Prescription opioid abuse remains an ongoing public health crisis, especially in orthopaedic surgery. The purpose of the present study is to analyze opioid-prescribing patterns and investigate risk factors for prolonged opioid use after common outpatient orthopaedic surgical procedures.

**Methods:**

After institutional review board approval, a review of 1,384 patients undergoing common elective outpatient orthopaedic procedures from January 2018 to June 2019 was conducted. Data on controlled substance prescriptions were obtained from the prescription drug monitoring program website. Statistical analysis was done to identify predictors for a second opioid prescription and prolonged opioid use (>6 months).

**Results:**

Over 10% (150/1,384) of patients were still using opioids beyond 6 months. Of the opioid exposed patients, 60.4% (174/288) filled at least 1 additional opioid prescription postoperatively, and 29.2% (84/288) filled prescriptions beyond 6 months, compared with 26.4% (289/1,096) and 6.0% (66/1,096) of opioid-naive patients, respectively. Following multivariate analysis, significant predictors for filling a second opioid prescription included preoperative opioid use, current smoker status, benzodiazepine use, psychiatric disorder, and advanced age.

**Conclusion:**

This study revealed risk factors for prolonged opioid use after orthopaedic surgery. Surgeons should be mindful of these risk factors and counsel patients regarding postoperative pain management.

Considerable efforts have been made to curtail opioid abuse by minimizing postoperative pain and opioid use with the introduction of preoperative counseling, multimodal pain strategies intraoperatively and postoperatively, and statewide online prescription drug monitoring programs (PDMPs). However, opioid abuse continues to remain a costly health burden.^1,2^ Opioid abuse and its health and societal ramifications cost the US healthcare system in excess of $78 billion annually.^3-5^ In 2018, 46,802 people died as a result of opioid overdose, with nearly one-third being from opioid prescriptions.^6^ Orthopaedic surgeons have been identified as high prescribers for opioids,^7^ and therefore, effective postoperative pain management protocols could help curtail the opioid epidemic.

The use of opioids after orthopaedic surgery has been explored in the literature. Studies have demonstrated that chronic opioid abuse leads to worse functional outcomes, delayed return to work, increased revision rates, and increased risk for postoperative infections.^8-10^ Preoperative opioid counseling and postoperative evidence-based opioid prescribing have shown to decrease opioid consumption while maintaining high satisfaction.^11-13^ In addition, multimodal pain management and interscalene blocks have proven effective means of postoperative pain control after upper extremity surgery.^14,15^ However, long-term opioid use after elective surgery is of particular concern, with Brummett et al^16^ identifying an approximate 6% rate of prolonged opioid use after major and minor general surgeries. Prolonged use is potentially representative of opioid abuse and dependency. Recently, studies in the arthroplasty literature have used PDMP systems to identify predictors of second postoperative opioid prescription rates and prolonged use beyond 6 months.^17,18^

It is relevant for orthopaedic surgeons to investigate factors that may place certain patients at risk for developing long-term opioid abuse after orthopaedic surgery. A patient developing chronic opioid abuse is not only detrimental to postoperative outcomes but also to a patient's overall health and to the healthcare system as a whole. The purpose of the present study is to investigate risk factors for prolonged opioid use after common elective outpatient orthopaedic surgical procedures using a state PDMP.

## Methods

After institutional review board approval, a retrospective review of 1,699 patients undergoing common outpatient orthopaedic procedures was conducted from January 2019 to June 2019 at a single institution. Inclusion criteria included patients at least 18 years old who underwent a surgical procedure from January 2018 to June 2019 at our institution, and patients were identified via a database query. Elective outpatient orthopaedic procedures selected for analysis included distal biceps repair (Current Procedural Terminology [CPT] 24342), rotator cuff repair (CPT 29827), anterior cruciate ligament reconstruction (CPT 29888), Achilles tendon repair (CPT 27650), hallux valgus correction (CPT 28295, 28296), and thumb basal joint arthroplasty (CPT 25447). Patients were excluded if they underwent more than one surgical procedure during the study period, or if they did not fill an initial postoperative opioid prescription. A cohort of 1,699 patients met the initial inclusion criteria, and of those, 1,383 patients could be identified in the PDMP system and filled their initial postoperative opioid prescription. Data obtained from institutional databases included demographic information, alcohol use, smoking status, and medical history. A history of psychiatric disorder was defined as having anxiety and/or depression.

The Pennsylvania PDMP website was used to identify controlled substance prescriptions filled by study subjects. States included in the Pennsylvania PDMP search include Pennsylvania, Arkansas, Connecticut, Delaware, Florida, Louisiana, Maine, Maryland, Massachusetts, Military Health System, Minnesota, New York, North Carolina, Ohio, Oklahoma, Rhode Island, South Carolina, Texas, Virginia, and West Virginia. The PDMP system has been previously validated and was proven to be reliable and accurate in controlled substance reporting.^19^ From 6 months preoperatively to 9 months postoperatively, all controlled substance prescriptions filled by each patient were recorded. This included opioids, benzodiazepines, stimulants, and nonbenzodiazepine sedative hypnotics. Additional information gathered from the PDMP system included prescription amount, prescription fill date, and the type of insurance that was used to purchase the controlled substance prescriptions. Patients who filled one controlled substance prescription during the 6-month preoperative period were considered opioid exposed to that controlled substance. Patients who did not fill any controlled substance prescriptions during the preoperative period were considered to be opioid naive to controlled substances. Postoperative dependent outcomes investigated included (1) filling an additional opioid prescription postoperatively and (2) prolonged opioid use greater than 6 months postoperatively. Patient characteristics and controlled substance use were analyzed to determine risk factors for these outcomes.

Continuous data are presented as mean (SD), and categorical data are presented as counts (%). Continuous and categorical data were initially analyzed with independent Student *t*-test and chi-square testing, respectively, to observe significant associations. The investigated variables were selected based on clinical relevance and analyzed for association with the primary outcomes. A stepwise multivariate regression for each primary outcome was then built using the significant variables. Variables with *P* > 0.25 were eliminated. Finally, the area under the curve (AUC) was calculated to determine the accuracy of the stepwise regression models for each outcome variable. Statistical significance was set at *P* < 0.05. All statistical analyses were done using R Studio (Version 3.6.3).

## Results

Overall, 1,383 patients were identified in the PDMP system and filled their initial postoperative opioid prescription. Patient mean age was 50.2 years (SD 16.9), with 787 men (56.9%) and 596 women (43.1%) (Table 1). With respect to preoperative controlled substance use, 20.8% used opioids, 11.1% used benzodiazepines, 4.8% used stimulants, 4.7% used nonbenzodiazepine sedative hypnotic medications, and 7.9% used more than one type of controlled substance. Additional demographic data are summarized in Table 1. The mean opioid prescription amount in total morphine milligram equivalents (MMEs) prescribed on the day of surgery was 290.6 MMEs (SD 221.8), which is equivalent to 39 tablets of 5 mg oxycodone. The type and number of procedures done are included in Table 2.

**Table 1:** Descriptive Statistics of Patients Who Filled an Additional Opioid Prescription and Who Had Prolonged Opioid Use >6 months Postoperatively

Variable	Total, N = 1,383	No Second Opioid Rx, N = 921	Filled a Second Opioid Rx, N = 462	*P*	No Prolonged Opioid Use, N = 1,234	Prolonged >6-mo Opioid Use, N = 149	*P*
Age (SD)	50.2 (16.9)	47.7 (17.2)	55.3 (15.1)	<0.001	49.2 (17.1)	58.3 (12.8)	<0.001
Sex				0.009			0.507
Female	596 (43.1%)	374 (40.6%)	223 (48.2%)		528 (42.8%)	69 (46.0%)	
Male	787 (56.9%)	547 (59.4%)	240 (51.8%)		706 (57.2%)	81 (54.0%)	
BMI (SD)	28.1 (5.5)	27.6 (5.16)	29.0 (5.90)	<0.001	27.8 (5.35)	30.2 (5.93)	<0.001
Preop opioid exposed	287 (20.8%)	114 (12.4%)	174 (37.6%)	<0.001	204 (16.5%)	84 (56.0%)	<0.001
Preop stimulant exposed	67 (4.8%)	40 (4.34%)	27 (5.83%)	0.278	60 (4.86%)	7 (4.67%)	1
Preop benzodiazepine exposed	153 (11.1%)	61 (6.62%)	92 (19.9%)	<0.001	119 (9.64%)	34 (22.7%)	<0.001
Preop sedative hypnotic exposed	65 (4.7%)	32 (3.47%)	33 (7.13%)	0.004	55 (4.46%)	10 (6.67%)	0.316
Use of >1 controlled substances	109 (7.9%)	30 (3.26%)	79 (17.1%)	<0.001	74 (6.00%)	35 (23.3%)	<0.001
Alcohol use	886 (68.9%)	589 (69.1%)	297 (68.4%)	0.871	791 (69.1%)	95 (66.4%)	0.573
Current smoker	124 (11.4%)	62 (8.60%)	62 (17.0%)	<0.001	99 (10.3%)	25 (20.7%)	0.001
Recreational drug use	27 (2.1%)	17 (2.01%)	10 (2.31%)	0.88	24 (2.11%)	3 (2.10%)	1
Psychiatric disorder	206 (16.0%)	94 (11.1%)	112 (25.7%)	<0.001	158 (13.8%)	48 (33.6%)	<0.001
Dementia	7 (0.6%)	4 (0.48%)	3 (0.70%)	0.695	5 (0.44%)	2 (1.42%)	0.178
Diabetes	88 (6.4%)	44 (4.78%)	44 (9.50%)	0.001	65 (5.27%)	23 (15.3%)	<0.001
Osteoporosis	67 (5.3%)	26 (3.08%)	41 (9.51%)	<0.001	43 (3.80%)	24 (16.8%)	<0.001
Sleep apnea	136 (10.6%)	72 (8.51%)	64 (14.8%)	0.001	105 (9.24%)	31 (21.8%)	<0.001
Stroke	12 (0.9%)	5 (0.59%)	7 (1.61%)	0.121	8 (0.71%)	4 (2.80%)	0.036
Insurance type				0.004			0.02
Nongovernment	1,234 (89.2%)	838 (91.0%)	397 (85.7%)		1,110 (90.0%)	125 (83.3%)	
Government	150 (10.8%)	83 (9.01%)	66 (14.3%)		124 (10.0%)	25 (16.7%)	

Data are represented as number (percentage) or mean (SD).

BMI = body mass index, Preop = preoperative, Rx = prescription

**Table 2:** Opioid Prescription Trends by Procedure Type and by Preoperative Opioid Exposure

Variable	Procedure Type								Opioid Exposed Versus Naive		
	Achilles, N = 128	ACLR, N = 433	BJA, N = 149	DBR, N = 62	HVC, N = 69	RCR, N = 542	Total, N = 1,383	*P*	Opioid, Naive, N = 1,096	Opioid Exposed, N = 287	*P*
Filled another Rx	20.3% (26)	21.7% (94)	45.0% (67)	22.6% (14)	29.0% (20)	44.5% (241)	33.4% (462)	<0.001	26.4% (289)	60.4% (174)	<0.001
Prolonged >6-mo use	3.9% (5)	3.2% (14)	16.8% (25)	14.5% (9)	11.6% (8)	16.2% (88)	10.8% (149)	<0.001	6.0% (66)	29.2% (84)	<0.001
Preop opioid usage	16.4% (21)	11.1% (48)	26.2% (39)	17.7% (11)	14.5% (10)	29.2% (158)	20.8% (287)	<0.001	—	100% (287)	—
Avg no. Rxs	1.2 (0.4)	1.4 (1.3)	3.3 (3.8)	2.2 (2.2)	2.4 (2.0)	3.0 (3.3)	2.6 (3.0)		—	2.6 (3.0)	—
Total MME received	149.1 (135.3)	378.3 (1,398.9)	2,459.0 (4,769.9)	1,128.4 (2,582.0)	1,116.5 (2,165.2)	3,244.9 (13,556.5)	2,276.9 (10,303.9)		—	2,276.9 (10,303.9)	—
Avg MME/Rx	127.2 (99.1)	159.6 (212.8)	628.4 (1,392.7)	259.6 (335.7)	266.4 (293.0)	418.6 (796.9)	371.0 (802.6)		—	371.0 (802.6)	—
Mean MME of the initial Rx	295.5 (136.0)	329.2 (209.1)	164.7 (159.0)	183.1 (90.9)	211.4 (69.4)	309.6 (223.2)	290.6 (221.8)		283.7 (170.0)	305.4 (297.6)	0.237
Postop opioid usage	20.3% (26)	21.7% (94)	45.0% (67)	22.6% (14)	29.0% (20)	44.5% (241)	33.4% (462)	<0.001			
Avg no. Rxs	1.6 (1.5)	1.6 (1.1)	3.4 (4.9)	2.2 (1.6)	2.3 (2.8)	3.1 (4.1)	2.7 (3.7)		1.6 (1.1)	4.5 (5.4)	<0.001
Total MME received	295.4 (352.5)	330.2 (610.1)	2,284.2 (6,403.0)	633.2 (867.8)	842.1 (2,512.7)	2,792.1 (11,736.4)	1,927.2 (8,895.2)		309.7 (283.1)	4,629.1 (14,149.2)	<0.001
Avg MME/Rx	169.9 (73.7)	178.9 (129.2)	282.3 (414.9)	221.7 (217.0)	194.7 (215.7)	365.2 (577.7)	292.5 (460.6)		186.0 (106.3)	470.5 (706.2)	<0.001

ACLR = anterior cruciate ligament reconstruction, Avg = average; BJA = basal joint arthroplasty, DBR = distal biceps repair, HVC = hallux valgus correction, MME = morphine milligram equivalents, Preop = preoperative, Postop = postoperative; RCR = rotator cuff repair, Rx = prescription

### Preoperatively Opioid Exposed Versus Naive

Within 6 months preoperatively, over 20% (287/1,383) of patients in this study used opioids, representing the opioid-exposed patients. These patients received an average of 2.6 prescriptions for a total of 2,276.9 MMEs (average of 371.0 MME/Rx) within 6 months before their surgery. Opioid-exposed patients were prescribed a slightly larger, but not statistically significant, opioid prescription on the day of their surgery for postoperative pain than did opioid-naive patients (305.4 MME versus 283.7 MME; *P* = 0.237). In addition, 60.6% (174/287) of opioid-exposed patients filled at least 1 additional opioid prescription postoperatively compared with 26.4% (289/1,096) of opioid-naive patients (*P* < 0.001). In addition, of the opioid-exposed patients, 29.3% (84/287) continued to fill opioid prescriptions beyond 6 months postoperatively compared with only 6.0% (66/1,096) of opioid-naive patients (*P* < 0.001). Overall, during the 12-month postoperative period, opioid-exposed patients filled significantly more opioid prescriptions (4.5 versus 1.6; *P* < 0.001), received significantly higher total amount in MMEs (4,629.1 versus 309.7; *P* < 0.001), and received larger prescriptions by average MME/Rx (470.5 versus 186.0; *P* < 0.001) than did opioid-naive patients (Table 2).

### Second Opioid Prescription

**Figure 1:**
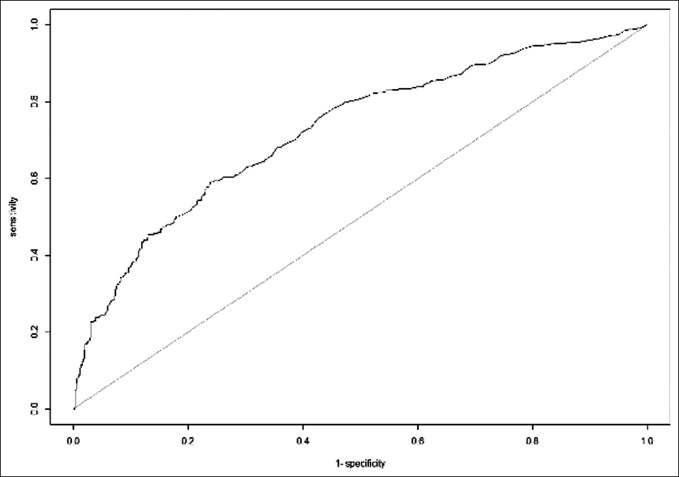
AUC curve for the regression model with an additional opioid prescription as the dependent outcome. The AUC value is 0.726. AUC = area under the curve

A second opioid prescription was filled by 33.4% (462/1,383) of patients. Patients who filled at least one additional opioid prescription postoperatively were prescribed a slightly larger initial postoperative opioid prescription than the patients who did not fill a second prescription; however, this difference was not statistically significant (294.9 MME versus 284.9 MME; *P* = 0.449) Following stepwise multivariate analysis, the following independent risk factors (in order of decreasing significance) were associated with filling an additional opioid prescription: preoperative opioid use (odds ratio [OR] 3.37; *P* < 0.001), current smoker status (OR 1.95; *P* = 0.002), history of psychiatric disorder (OR 1.81; *P* = 0.002), preoperative benzodiazepine use (OR 1.76; *P* = 0.015), and older age (OR 1.02; *P* ≤ 0.001) (Table 3). The AUC value, which represents the accuracy of a regression model, for the regression predicting an additional opioid prescription was 0.726 (Figure 1).

**Table 3:** Multivariate Regression With an Additional Opioid Prescription as the Dependent Outcome

Variable	Odds Ratio (95% CI)	*P*
Preop opioid use	3.37 (2.44–4.66)	<0.001
Preop benzodiazepine use	1.76 (1.11–2.80)	0.015
Age	1.02 (1.01–1.03)	<0.001
Smoker	1.95 (1.29–2.96)	0.002
Psychiatric disorder	1.81 (1.25–2.62)	0.002

CI = confidence interval, Preop = preoperative

### Prolonged Opioid Use >6 Months

**Figure 2:**
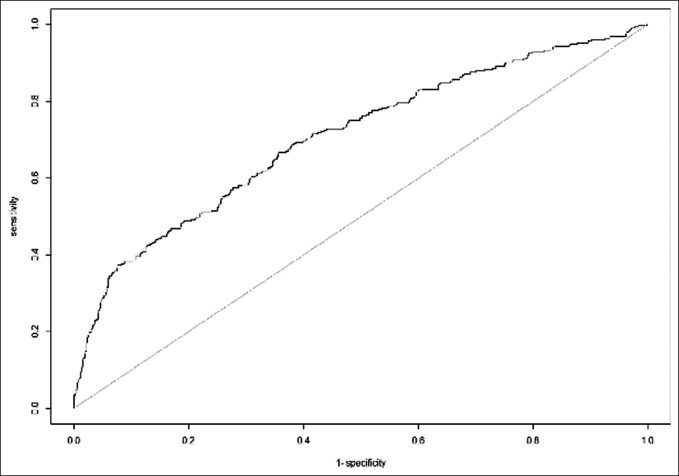
AUC curve for the regression model with prolonged >6-month opioid use as the dependent outcome. The AUC value for this curve is 0.706. AUC = area under the curve

Prolonged opioid use >6 months postoperatively was observed in 10.8% (149/1,383) of patients. Following stepwise multivariate regression analysis, the following independent variables (in order of decreasing significance) were associated with prolonged >6-month opioid use: preoperative opioid use (OR 5.91; *P* < 0.001), history of sleep apnea (OR 2.17; *P* = 0.006), history of osteoporosis (OR 2.14; *P* = 0.030), current smoker status (OR 2.01; *P* = 0.011), and advanced age (OR 1.03; *P* = 0.002) (Table 4). The AUC value for this regression model predicting prolonged >6-month opioid use was 0.706 (Figure 2).

**Table 4:** Multivariate Regression With Prolonged >6-Month Opioid Use as the Dependent Outcome

Variable	Odds Ratio (95% CI)	*P*
Preop opioid use	5.91 (3.89–9.06)	<0.001
BMI	1.03 (0.99–1.07)	0.059
Age	1.03 (1.01–1.04)	0.002
Government insurance	0.60 (0.31–1.14)	0.128
Smoker	2.01 (1.16–3.41)	0.011
Osteoporosis	2.14 (1.06–4.22)	0.030
Sleep apnea	2.17 (1.24–3.75)	0.006

BMI = body mass index, CI = confidence interval, Preop = preoperative

### Procedure Type

The type and number of procedures done are included in Table 2. Mean total MMEs of the initial postoperative opioid prescription for specific procedure types ranged from 164.7 MMEs (SD 159.0) (equivalent of 21 tablets of 5 mg oxycodone) for basal joint arthroplasty (BJA) to 329.2 MMEs (SD 209.1) (equivalent of 44 tablets of 5 mg oxycodone) for anterior cruciate ligament reconstruction (Table 2). For all procedures, there were more patients who filled additional opioid prescriptions postoperatively than there were patients who used opioids preoperatively. Significantly more rotator cuff repair (RCR) patients (29.2%) and BJA patients (26.2%) used opioids preoperatively compared with the other procedures (*P* < 0.001). Postoperatively, significantly more BJA patients (45.0%) and RCR patients (44.5%) filled a second opioid prescription compared with the other procedures (*P* < 0.001). Prolonged >6-month opioid use postoperatively was observed in significantly more BJA patients (16.8%), RCR patients (16.2%), and distal biceps repair patients (14.5%) than the other procedures (*P* < 0.001). Postoperatively, BJA and RCR patients received more opioid prescriptions and a higher total MME amount when compared with all other procedures (Table 2).

## Discussion

In an effort to combat the national opioid epidemic, healthcare providers and researchers continue to investigate different strategies to manage postsurgical pain.^1,2,14,20^ This study identifies independent risk factors for filling an additional opioid prescription and prolonged opioid use after common elective outpatient orthopaedic surgical procedures. As cited in previous studies, preoperative opioid exposure was a significant risk factor for prolonged opioid use postoperatively. In addition, a history of smoking and advanced age were risk factors for both filling an additional opioid prescription and prolonged opioid use. To our knowledge, this study represents the largest sample of PDMP data currently published in the literature; our hope is that our findings can be used to aid in identifying patients at risk for prolonged opioid use and prevent possible addiction.

Our study identified a long-term use of opioids postoperatively, as defined as >6 months, to be 10.8%, with a rate of 6.0% in the opioid-naive cohort. This is consistent with other studies within the literature. Jiang et al^7^ found that chronic opioid use (>3 months) prevalence to be on average 9.2% across different surgical subspecialties in opioid-exposed and -naive patients. In a large 2017 insurance claims database study, Brummett et al16 found that opioid-naive patients undergoing minor and major general surgery procedures had rates of new persistent opioid use (>3 months) ranging from 5.9% to 6.5%. Similarly, in a retrospective study of 35,817 opioid-naive patients undergoing cardiac surgery, Brown et al^21^ found new and persistent opioid use (90 to 180 days) to be 10.2 and 8.1% after undergoing coronary artery bypass grafting and heart valve procedures, respectively. Within the orthopaedic literature, Khazi et al^22^ found a rate of prolonged use in opioid-naive patients of 6.3% at 3 months after undergoing total shoulder arthroplasty compared with 46% in opioid-exposed patients. Following total hip arthroplasty, Tan et al^17^ reported rates of prolonged opioid use >6 months of 25.0% in opioid-exposed patients compared with 2.7% of opioid-naive patients.

Preoperative opioid use has been examined in the orthopaedic literature. Previous studies have reported rates of preoperative opioid exposure, ranging from 17.4% to 34.9%, and its effect on postoperative opioid use.^9,23,24^ Similarly, we report a preoperative opioid use rate of 20.8%. Preoperative opioid exposure represents a significant risk for prolonged opioid use, as we observed that over 60% of these patients filled an additional opioid prescription and approximately 30% continued to fill opioid prescriptions more than 6 months postoperatively. Furthermore, over one-quarter (26.4%) of opioid-naive patients filled a second opioid prescription. We observed that preoperative opioid-exposed patients filled significantly more prescriptions, received significantly more total MMEs, and received significantly larger prescriptions on average postoperatively than did preoperative opioid-naive patients. Interestingly, preoperative benzodiazepine use and a history of anxiety and/or depression were only found to be risk factors for filling a second opioid prescription, but not with prolonged opioid use. Also, sleep apnea, often an indication of poor overall health, was significantly associated with prolonged opioid use, but not with filling a second prescription.

Patient risk factors and characteristics for chronic opioid use have also been reported in the orthopaedic literature. Jiang et al,^7^ in a large database study of 79,123 patients, investigated chronic opioid use (>3 months postoperatively) rates across different surgical subspecialties, with the highest prevalence in orthopaedic surgery at 23.8%, followed by neurosurgery (18.7%), and gastrointestinal surgery (14.4%). Among potential risk factors, investigators found underweight body mass index (OR 2.08), age 50 to 59 years (OR 2.06), and age 40 to 49 years (OR 2.01) to be the highest risk factors for chronic use (*P* < 0.0001). However, preoperative opioid status was not taken into account. Although much of the current literature focuses on patient-specific risk factors for developing prolonged opioid use postoperatively, fewer studies have investigated how nonpatient factors may contribute to these adverse outcomes. Nonpatient factors that could theoretically contribute to patients developing opioid dependence could include postoperative opioid prescribing protocols, refill patterns, and physician-specific prescribing trends. How individual surgeon characteristics, such as training background, experience, and beliefs, contribute to opioid-prescribing patterns is a topic that warrants further investigation.

The orthopaedic literature has consistently associated chronic opioid usage with poor outcomes. Bell et al,^9^ in a large retrospective database study of 23,754 patients, found preoperative opioid usage to be an independent risk factor in the development of periprosthetic joint infection in patients undergoing primary total joint arthroplasty (OR 1.63, *P* = 0.005). Similarly, Bedard et al, in a retrospective registry study of 17,695 patients, reported that patients who were exposed to opioids within 3 months of primary total hip arthroplasty had an increased risk of early revision compared with those who were not exposed to opioids preoperatively (1.2% versus 0.7%, *P* < 0.001). Furthermore, they also found that a preoperative diagnosis of anxiety/depression had an even greater risk of early revision (1.9% versus 0.8%, *P* = 0.006).^8^ Preoperative and chronic opioid exposure may also signal a lower baseline level of functioning and potential for recovery. Kazmers et al^23^ assessed the effect of baseline opioid usage on the functional and psychological outcomes in upper extremity patients and found that patients with chronic opioid exposure demonstrated significantly worse scores on all patient-reported outcomes (Patient-Reported Outcomes Measurement Information System [PROMIS]) and functional outcomes (PROMIS and Quick Disabilities of the Arm, Shoulder, and Hand [QDASH]).

Preventive measures in the form of preoperative pain counseling have proven effective in reducing opioid usage perioperatively.^2,20^ Vincent et al,^2^ in a prospective randomized controlled trial of 131 patients, found that patients undergoing upper extremity surgery who received preoperative opioid counseling on postoperative opioid consumption consumed significantly fewer pills than the control group who did not receive counseling (11.8 pills versus 17.4 pills, *P* = 0.007), with no difference in pain scores at any time point. Similarly, a systematic review and meta-analysis by Gazendem et al^20^ concluded preoperative pain counseling effective in overall opioid consumption.

This study is not without limitation. First, the retrospective nature of the study lends itself to the inherent bias of retrospective study design along with the reliance of proper documentation. However, every effort was made to confirm accuracy of data. Second, this study only evaluated filled opioid prescriptions, which does not always correlate with patient consumption. Third, it is possible that patients may be obtaining additional opioids through other avenues not recorded in the PDMP system. Thus, it is possible that consumption is underreported in our series. Fourth, adjuvant use of multimodal nonopioid pain regimens was not evaluated. Future investigations should examine the potential opioid-sparing role of multimodal pain regimens. Finally, our analysis was limited to outpatient procedures across several subspecialties within orthopaedic surgery, and thus, our findings may not be generalizable to all orthopaedic procedures.

Preoperative opioid use and a history of current smoking, sleep apnea, osteoporosis, and advanced age were significant predictors for second opioid prescription and prolonged use beyond 6 months after outpatient orthopaedic surgery. It is our hope that orthopaedic surgeons may use these characteristics to identify and treat patients at higher risk for prolonged opioid.
